# The Abridgment and Relaxation Time for a Linear Multi-Scale Model Based on Multiple Site Phosphorylation

**DOI:** 10.1371/journal.pone.0133295

**Published:** 2015-08-11

**Authors:** Shuo Wang, Yang Cao

**Affiliations:** Department of Computer Science, Virginia Tech, Blacksburg, VA, United States of America; University of Edinburgh, UNITED KINGDOM

## Abstract

Random effect in cellular systems is an important topic in systems biology and often simulated with Gillespie’s stochastic simulation algorithm (SSA). Abridgment refers to model reduction that approximates a group of reactions by a smaller group with fewer species and reactions. This paper presents a theoretical analysis, based on comparison of the first exit time, for the abridgment on a linear chain reaction model motivated by systems with multiple phosphorylation sites. The analysis shows that if the relaxation time of the fast subsystem is much smaller than the mean firing time of the slow reactions, the abridgment can be applied with little error. This analysis is further verified with numerical experiments for models of bistable switch and oscillations in which linear chain system plays a critical role.

## Introduction

With the rapid development in systems biology, biological models have become more and more complex. In many complex biochemical systems, stochastic effects in cells are of particular concern because some species, such as genes and RNAs, present with small copy numbers in those systems. In order to study the stochastic effects, stochastic biochemical models have been built and simulated. Gillespie’s stochastic simulation algorithm (SSA) [1, 2] is one of the most important stochastic methods. However, the computational cost of the SSA can be very high, particularly for systems with the multi-scale feature, which highlights the scale differences among reactions: Some reactions fire much faster than others, and those fast reactions often quickly reach equilibrium. Since the SSA tracks every reaction firing, in an SSA simulation most computational costs are spent on fast reactions. On the other hand, slow reactions may be more important because very often they drive the dynamics of a system when fast reactions are in equilibrium.

Several approximation methods have been proposed to utilize this multiscale feature. The key idea of these approximation methods is to approximate the propensities of slow reactions by taking account of the effect of fast reactions while avoiding full simulation of them. A common practice is to reduce a complex system to a simpler model that well approximates the original system. A well-known example of such a reduction is the Michaelis-Menten equation of the enzyme-substrate reactions, which has played an important role in biochemistry. Following Gillespie et al. [3], we will call such a reduction abridgment. An abridgment replaces a reaction network with a network with fewer reactions and chemical species. An obvious advantage of an abridgment is the reduction in the numbers of reactions and species that we have to deal with. Another advantage might be speeding up the numerical simulation. But we also have to realize the drawback. An abridgment is still an approximation by nature. We have to study conditions that an abridgment is valid. In Gillespie et. al. [3], a detailed discussion was given on the condition that the system

$$\begin{array}{c} S_{1}\overset{c_{1}}{\underset{c_{2}}{⇌}}S_{2}\overset{c_{3}}{\to }S_{3} \end{array}$$ (1)

can be reduced to a simple one

$$\begin{array}{c} S_{1}\overset{c}{\to }S_{3}, \end{array}$$ (2)

where *c* is given some “suitable” value depending on *c*_1_, *c*_2_, and *c*_3_. It was concluded that the abridgment is valid if and only if one of the following conditions is satisfied:

$$\begin{array}{c} c_{2}\gg c_{1},\;c_{3}\gg c_{1},\;c_{1}\gg c_{3},\;\;\text{or}\;\;c_{2}\gg c_{3}. \end{array}$$ (3)

In this paper we consider a more general problem: Given a linear chain reaction model

$$\begin{array}{c} S_{1}\overset{f_{1}}{\underset{b_{1}}{⇌}}S_{2}\overset{f_{2}}{\underset{b_{2}}{⇌}}\cdots \overset{f_{n-1}}{\underset{b_{n-1}}{⇌}}S_{n}\overset{f_{n}}{\to }S_{n+1}, \end{array}$$ (4)

where rate constants *f*_*i*_’s and *b*_*i*_’s may depend on other variables in the system but not on *S*_*i*_’s, what is the condition that this system can be reduced to

$$\begin{array}{c} S_{1}\overset{c}{⟶}S_{n+1}, \end{array}$$ (5)

where *c* depends on the values of *f*_*i*_’s and *b*_*i*_’s?

**Fig 1:**
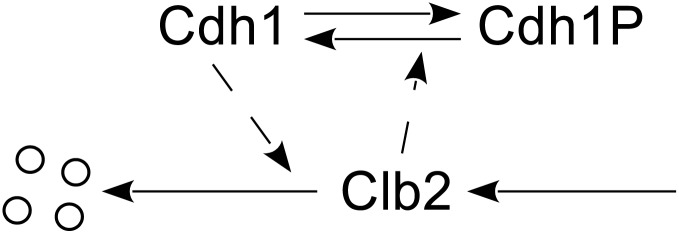
An example of bistable switch in the cell cycle model. Each dashed line indicates the enzyme species on one enzyme-substrate reaction.

System (4) is not just a simple generalization of (1). It represents an important module in biological systems as well. For example, one of the most interesting and well studied biological systems is the cell cycle model, which aims to accurately model the repetitive sequence of events that a cell grows, replicates its components, and divides into two daughter cells. A key component in the cell cycle model is bistable switch, such as the one shown in Fig 1, where Cdh1 and Clb2 are regulative proteins inhibiting each other to form a positive feedback loop. One typical setting of the two enzyme-substrate reactions can be the Michaelis-Menten rate law equation, which assumes that the concentration of the enzyme species be much smaller than that of the substrate. However, this assumption may not always be true in cell cycle models. Particularly in this example, since Cdh1 and Clb2 serve as enzyme to each other. There is no way that the aforementioned assumption will be valid for both directions. Thus different kinetic laws have to be adopted to solve this dilemma. Elementary reactions [1, 2] are particularly desired in stochastic modeling and simulations.

**Fig 2:**
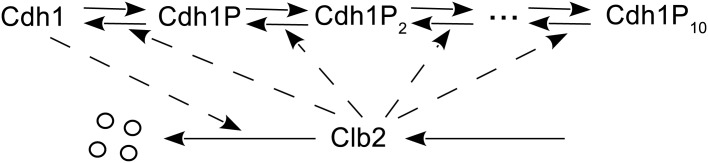
A bistable switch converted from the model in Fig 1 based on multiple site phosphorylation. Cdh1 is assumed to have 11 phosphorylation levels, as described as Cdh1 (the unphosphorylated form) and Cdh1P through Cdh1P_10_.

One effort to solve this dilemma is to break down simple switch systems constructed with Michaelis-Menten rate laws into elementary reaction rate laws. Kar et al. [4] managed to replace a simple version of the budding yeast cell cycle model with a quite complex network of elementary chemical reactions with corresponding rate laws. Another interesting discovery by Qu et al. [5] shows that, modeling the regulative protein with multiple site phosphorylation may also produce the “nonlinearity” in the system and generate bistable switch behavior. The idea is to assume that the regulative protein have multiple phosphorylation levels, which enable it to undergo multiple (de)phosphorylation reactions between different levels. Fig 2 shows an example of a bistable switch based on this idea. It manages to achieve the required nonlinearity with only *elementary reactions* at the cost of a relatively more complicated network. Note that it is not clear in reality which rate law does apply in molecular regulatory networks. It might be neither Michaelis-Menten nor elementary reaction rate laws. However, multisite phosphorylation based on elementary reaction rate laws does provide a convenient mechanism that can be easily modeled either deterministically or stochastically. This idea was originally proposed by Kapuy et al. [6], who proposed that multisite phosphorylation sequences may be modeled by elementary reaction rate laws and are suitable for both deterministic and stochastic simulations. Later Barik et al. [7] developed a model of yeast cell cycle regulation based on multiple site (de)phosphorylation reactions and elementary reaction rate laws. Their model was compared with experimental measurements [8]. However, this modeling technique presents challenges for current stochastic simulation algorithms. Most reaction firings are for the (de)phosphorylation reactions, which typically result in frequent but “back-and-forth” changes that cancel each other’s effect. The overall stochastic characteristics of the model is mostly driven by other slow reactions. It would be ideal if one could reduce the system in Fig 2 to the simpler version in Fig 1. Studying reduction condition for the system (4) will help us achieve this goal.

Another biochemical example of system (4) comes from one dimensional reaction-diffusion system, where diffusion is usually much faster than reaction. Given a particular partition (with *n* linear bins), the diffusion can be formulated as a chain reaction. Suppose the diffusion rates are uniform and the bins are equally spaced, if we can reduce system (4) to the simple system (1), then we basically reduce a spatially inhomogeneous system to a spatially homogeneous system. Thus in this case the condition for the reduction of system (4) is also related to the condition for the famous “well-stirred” assumption given in Gillespie’s pioneering paper [1, 2].

In this paper we present an error analysis of abridgment based on comparison of the first exit time. Although our analysis focuses on the chain reaction system (4), the result can be extended to more complicated linear systems. Details of the analysis is given in the Methods section and we demonstrate one specific application on bistable switch and oscillation models in the Results section.

## Methods

### Background

In this section, we briefly review the simulation methods: SSA and SQSSA/ssSSA.

#### SSA

Suppose the system involves *N* molecular species {*S*_1_, …, *S*_*N*_}. The state vector is denoted by *X*(*t*) = (*X*_1_(*t*), …, *X*_*N*_(*t*)), where *X*_*i*_(*t*) is the number of molecules of species *S*_*i*_ at time *t*. *M* reaction channels {*R*_1_, …, *R*_*M*_} are involved in the system. Assume that the system is well stirred and is in thermal equilibrium. The dynamics of reaction channel *R*_*j*_ is characterized by the *propensity function* *a*_*j*_ and the *state change vector* *ν*_*j*_ = (*ν*_1*j*_, …, *ν*_*N*,*j*_): *a*_*j*_(*x*)*dt* gives the probability that one *R*_*j*_ reaction will occur in the next infinitesimal time interval [*t*, *t* + *dt*), and *ν*_*ij*_ gives the change in the *S*_*i*_ molecule population induced by one *R*_*j*_ reaction.

The dynamics of the system can be simulated by the SSA. With *X*(*t*) = *x*, let $a_{0}(x)=\sum_{j=1}^{M}a_{j}(x)$. In each step, the SSA generates two random numbers *r*_1_ and *r*_2_ in *U*(0,1), the uniform distribution in the interval (0,1). The time for the next reaction to occur is given by *t*+*τ*, where *τ* is given by

$$\begin{array}{c} \tau =\frac{1}{a_{0}\left(x\right)}\log(\frac{1}{r_{1}}). \end{array}$$ (6)

The index *j* for the next reaction is given by the smallest integer satisfying

$$\begin{array}{c} \sum_{l=1}^{j}a_{l}\left(x\right)>r_{2}a_{0}\left(x\right). \end{array}$$ (7)

The system states are updated by *X*(*t*+*τ*) = *x*+*ν*_*j*_. The simulation proceeds to the next occurring time, until it reaches the final time or other stop criteria.

#### SQSSA/ssSSA

The stochastic quasi–steady–state approximation (SQSSA) [9] and slow–scale SSA (ssSSA) [10, 11] were proposed to reduce the size of a stochastic biological model and to improve the stochastic simulation efficiency. The ssSSA is based on the partial equilibrium (PE) assumption, while the SQSSA is based on the quasi steady state (QSS) assumption. The PE and QSS assumptions are both important multiscale features in biochemical systems. PE refers to the situation where some reactions fire much faster than others and the corresponding subsystem reaches a partial equilibrium state [10]. QSS refers to the situation where some state variables fluctuate very quickly around their quasi steady states [9]. If a subsystem is at PE, then all its involved species are in QSS.

To implement the SQSSA or ssSSA method, a system is partitioned into fast and slow subsystems, and the state variables are partitioned into fast and slow variables. The fast subsystem is assumed at an equilibrium state, while the state variables in the fast subsystem are in QSS. Then the simulation procedure is very similar to the original SSA procedure, except that in each simulation step, a set of algebraic equations is solved to find the QSS of all fast variables. Then these fast variables are considered (temporary) constant parameters in the slow subsystem and the SSA procedure is applied only the slow subsystem. Because the number of reaction firings in the slow subsystem is much less than the one for the fast subsystem, high simulation efficiency is achieved. However, if the time scale difference is not large enough, the QSSA method may lead to large errors. On the other hand, even if timescale separation holds, SQSSA may also lead to significant errors. Thomas et al. studied this problem in the work [12].

### Analysis for the Chain Reaction System with One Particle

Error analysis for abridgment in the stochastic regime is a subtle business [3]. The justification in SQSSA [9] and ssSSA [10, 11] were both based on distribution distance of ensembles collected with multiple runs of the original SSA and the corresponding approximation methods. But that is not good enough, in Thomas et. al [13], it was shown that “reduced master equation approach can overestimate the variance of the fluctuations by as much as ∼ 30%. Thus a more rigorous error analysis is needed when dealing with abridgment in the stochastic regime. In Gillespies et. al’s work [3], a novel error analysis method was proposed based on the first exit time analysis. To study the condition so that reaction (2) is an accurate reduction of system (1) with given populations of *S*_1_ and *S*_2_, the distribution of the first exit time, the time when the first *S*_3_ is generated, was analyzed for both Eqs (1) and (2). For the abridgment to be valid, the two distributions should be very close. In the simple system (2), the first exit time has an exponential distribution. Thus the abridgment is accurate only when the distribution of the first exit time for system (1) is close to an exponential distribution with a mean value close to the one from the simple system (2). Otherwise, production of *S*_3_ in (2) significantly differs from the one in (1) and as a result abridgment cannot be applied. Conditions (3) are thus derived based on this analysis [3].

In this paper, we follow a strategy similar as what was utilized in Gillespie et al. [3] and study the general case (4). In order to study the chain reaction system (4), we start with a simple case. Suppose there is only one particle starting at state *S*_1_. We will study the distribution function of the exit time *T*, the first time that this particle becomes *S*_*n*+1_. Following the analysis of Theorem 1 in Gillespie et. al [3], if the system (4) can be reduced to (5), the distribution for *T* should follow an exponential distribution, i.e.

$$\begin{array}{c} cdf\left(T;t\right)=\text{Prob}\left(T<t\right)=1-e^{-ct}, \end{array}$$ (8)

or equivalently

$$\begin{array}{c} \text{Prob}\left(T\geq t\right)=e^{-ct}. \end{array}$$ (9)

Denote

$$\begin{array}{c} p_{i}\left(t\right)=\text{Prob}\left(X_{i}\left(t\right)=1\right),\;\;\text{for}\;i=1,\ldots ,n+1, \end{array}$$ (10)

and let ℙ(*t*) = (*p*_1_(*t*), …, *p*_*n*_(*t*))^*T*^. Note that for this one particle, it takes one and only one state at any given time. Thus we always have

$$\begin{array}{c} \sum_{i=1}^{n+1}p_{i}\left(t\right)=1. \end{array}$$ (11)

When *X*_*n*+1_(*t*) = 1, it implies that the exit time *T* < *t*. Thus we have

$$\text{Prob}\left(T<t\right)=p_{n+1}\left(t\right)=1-\sum_{i=1}^{n}p_{i}\left(t\right),$$

or equivalently,

$$\begin{array}{c} \text{Prob}\left(T\geq t\right)=\sum_{i=1}^{n}p_{i}\left(t\right)=1^{T}\mathbb{P}\left(t\right), \end{array}$$ (12)

where **1** = (1, …, 1)^*T*^. Therefore, in order to obtain the analytic distribution function of *T* we need to study the equation for ℙ(*t*). We can write down the chemical master equation for ℙ as:

$$\begin{array}{c} \frac{d\mathbb{P}}{dt}=A\mathbb{P}, \end{array}$$ (13)

where

$$\begin{array}{c} A=[\begin{array}{cccccc} -f_{1} & b_{1} & 0 & 0 & \cdots & 0\\ f_{1} & -(b_{1}+f_{2}) & b_{2} & 0 & \cdots & 0\\ 0 & f_{2} & -(b_{2}+f_{3}) & b_{3} & \ddots & 0\\ 0 & \ddots & \ddots & \ddots & \ddots & 0\\ 0 & \cdots & 0 & f_{n-2} & -(b_{n-2}+f_{n-1}) & b_{n-1}\\ 0 & \cdots & 0 & 0 & f_{n-1} & -(b_{n-1}+f_{n}) \end{array}], \end{array}$$ (14)

with the initial condition ℙ(0) = (1, 0, …, 0)^*T*^.

*A* has *n* negative eigenvalues [14, 15]. We denote them as *λ*_1_ ≤ *λ*_2_ ≤ … ≤ *λ*_*n*_ < 0, and the corresponding eigenvectors as *ξ*_1_, …, *ξ*_*n*_. For the linear ODE (13), we can formulate its analytic solution as $\mathbb{P}(t)=\sum_{i=1}^{n}c_{i}e^{\lambda_{i}t}\xi_{i}$, where *c*_*i*_’s are determined by the initial state. Thus $\text{Prob}(T\geq t)={\text{1}}^{T}\mathbb{P}(t)=\sum_{i=1}^{n}c_{i}{\text{1}}^{T}\xi_{i}e^{\lambda_{i}t}$, which can be well approximated by an exponential function as shown in (9) if the following condition is satisfied:

$$\begin{array}{c} |\lambda_{n}\left|\ll \right|\lambda_{i}|,\;\;\text{for}\;\text{all}\;1\leq i\leq n-1. \end{array}$$ (15)

Moreover, when the condition (15) is satisfied, the reaction rate for the reduced system (5) is *c* = ∣*λ*_*n*_∣.

Condition (15) is a sufficient condition for the abridgment to be valid. But how is the condition (15) related to the fast/slow partitioning of the original system (4)? Next we will reveal a connection between the condition (15) and the relaxation time of the fast subsystem.

### Fast Subsystem and its Relaxation Time

We assume that reactions in system (4) are all fast except the last step, and then consider the fast subsystem:

$$\begin{array}{c} S_{1}\overset{f_{1}}{\underset{b_{1}}{⇌}}S_{2}\overset{f_{2}}{\underset{b_{2}}{⇌}}\cdots \overset{f_{n-1}}{\underset{b_{n-1}}{⇌}}S_{n}, \end{array}$$ (16)

with only one particle in the system. For this subsystem, we denote its state variables as ${\hat{X}}_{i}(t),i=1,\ldots ,n$. Let

$$\begin{array}{c} {\hat{p}}_{i}\left(t\right)=\text{Prob}\left({\hat{X}}_{i}\left(t\right)=1\right),\;\text{for}\;i=1,\ldots ,n, \end{array}$$ (17)

and $\hat{\mathbb{P}}(t)={\left({\hat{p}}_{1}(t),\ldots ,{\hat{p}}_{n}(t)\right)}^{T}$. We have a corresponding CME for $\hat{\mathbb{P}}$:

$$\begin{array}{c} \frac{d\hat{\mathbb{P}}}{dt}=B\hat{\mathbb{P}}, \end{array}$$ (18)

where

$$\begin{array}{c} B=[\begin{array}{cccccc} -f_{1} & b_{1} & 0 & 0 & \cdots & 0\\ f_{1} & -(b_{1}+f_{2}) & b_{2} & 0 & \cdots & 0\\ 0 & f_{2} & -(b_{2}+f_{3}) & b_{3} & \ddots & 0\\ 0 & \ddots & \ddots & \ddots & \ddots & 0\\ 0 & \cdots & 0 & f_{n-2} & -(b_{n-2}+f_{n-1}) & b_{n-1}\\ 0 & \cdots & 0 & 0 & f_{n-1} & -(b_{n-1}) \end{array}]. \end{array}$$ (19)

Similar to matrix *A*, *B* has *n* − 1 negative eigenvalues and one zero eigenvalue [14, 15]. Therefore, we can denote *B*’s eigenvalues as ${\hat{\lambda }}_{1}\leq {\hat{\lambda }}_{2}\leq \ldots \leq {\hat{\lambda }}_{n-1}<{\hat{\lambda }}_{n}=0$, and the corresponding eigenvectors as *η*_1_, …, *η*_*n*_. Define

$$\begin{array}{c} T_{relax}=\max_{1\leq i\leq n-1}\frac{1}{|{\hat{\lambda }}_{i}|}=\frac{1}{|{\hat{\lambda }}_{n-1}|}. \end{array}$$ (20)

*T*_*relax*_ is called the relaxation time of the fast subsystem (16). It is an important characteristic for the subsystem (16). The relaxation time represents the time scale for a system to “relax” to its steady state. It also can be viewed as the time scale after which the fast subsystem will “forget” a previous perturbation. To see this, we study the solution of the Eq (18), given by

$$\begin{array}{c} \hat{\mathbb{P}}=\sum_{1}^{n}{\hat{c}}_{i}e^{{\hat{\lambda }}_{i}t}\eta_{i}={\hat{c}}_{n}\eta_{n}+\sum_{1}^{n-1}{\hat{c}}_{i}e^{{\hat{\lambda }}_{i}t}\eta_{i}. \end{array}$$ (21)

When $|{\hat{\lambda }}_{i}t|\gg 1$ for 1 ≤ *i* ≤ *n* − 1, $\hat{\mathbb{P}}\to {\hat{\mathbb{P}}}^{*}={\hat{c}}_{n}\eta_{n}$. In other words, when *t* ≫ *T*_*relax*_, $\hat{\mathbb{P}}$ reaches its equilibrium state ${\hat{\mathbb{P}}}^{*}$.

To see the connection between the relaxation time and the abridgment, let *E* = *A* − *B*. *E*’s elements are all zero except the last diagonal element −*f*_*n*_. *E* can be considered as a rank one perturbation matrix. Thus for the eigenvalues *λ*_*i*_’s and ${\hat{\lambda }}_{i}$’s, we have (page 101 in Wilkinson [16])

$$\begin{array}{c} 0\leq {\hat{\lambda }}_{i}-\lambda_{i}\leq f_{n},\;\;\text{for}\;i=1,\ldots ,n. \end{array}$$ (22)

Thus ∣*λ*_*n*_∣ ≤ *f*_*n*_ and $|\lambda_{i}|\geq |{\hat{\lambda }}_{i}|$ for *i* = 1, …, *n* − 1. So condition (15) will be valid if the following condition is satisfied:

$$\begin{array}{c} f_{n}\ll \min_{1\leq i\leq n-1}|{\hat{\lambda }}_{i}\left|=\right|{\hat{\lambda }}_{n-1}|. \end{array}$$ (23)

The condition (23) has a special feature. On the left side, *f*_*n*_ is the reaction rate for the slow reaction, while on the right side, $|{\hat{\lambda }}_{n-1}|$ is a characteristic for the fast subsystem. We can also rewrite condition (23) as its equivalent condition:

$$\begin{array}{c} T_{relax}=\frac{1}{|{\hat{\lambda }}_{n-1}|}\ll \frac{1}{f_{n}}, \end{array}$$ (24)

where $\frac{1}{f_{n}}$ is the mean firing time for the slow reaction.

Based on the previous analysis, we can conclude that if the condition (23) is satisfied, the abridgment from system (4) to system (5) is valid with the reaction rate *c* = ∣*λ*_*n*_∣.

### Reaction Rate for the Reduced System

The above section reveals the connection between the abridgment and the relaxation time. But from a practical point of view, we still need to calculate the reaction rate *c* for the reduced system (5). Moreover, we need to calculate or estimate *T*_*relax*_.

First, we need to solve the equilibrium state ${\hat{\mathbb{P}}}^{*}$ of the system (16), which satisfies

$$\begin{array}{c} B{\hat{\mathbb{P}}}^{*}=0. \end{array}$$ (25)

*B* is singular. (25) does not uniquely give a solution for the equilibrium state. We need to realize that for system (16),

$$\begin{array}{c} \sum_{i=1}^{n}{\hat{p}}_{i}=1. \end{array}$$ (26)

We apply (26) and substitute ${\hat{p}}_{n}$ to obtain a nonsingular matrix. From (26) we have

$$\begin{array}{c} {\hat{p}}_{n}=1-\sum_{i=1}^{n-1}{\hat{p}}_{i}. \end{array}$$ (27)

Let $\hat{\mathbb{P}}$ denote the vector $\left\{{\hat{p}}_{1},\ldots ,{\hat{p}}_{n-1}\right\}$. We then have the equation

$$\begin{array}{c} \frac{d\hat{\mathbb{P}}}{dt}=\tilde{B}\hat{\mathbb{P}}+y, \end{array}$$ (28)

where

$$\tilde{B}=[\begin{array}{cccccc} -f_{1} & b_{1} & 0 & 0 & \cdots & 0\\ f_{1} & -(b_{1}+f_{2}) & b_{2} & 0 & \cdots & 0\\ 0 & f_{2} & -(b_{2}+f_{3}) & b_{3} & \ddots & 0\\ \vdots & \ddots & \ddots & \ddots & \ddots & \vdots \\ 0 & \cdots & 0 & f_{n-3} & -(b_{n-3}+f_{n-2}) & b_{n-2}\\ -b_{n-1} & \cdots & -b_{n-1} & -b_{n-1} & f_{n-2}-b_{n-1} & -(b_{n-2}+f_{n-1}+b_{n-1}) \end{array}]$$

and *y* = (0, …, 0, *b*_*n*−1_)^*T*^. One can verify that $\tilde{B}$ is nonsingular and the solution *β* of the equilibrium equation

$$\begin{array}{c} \tilde{B}\hat{\mathbb{P}}+y=0 \end{array}$$ (29)

gives the unique stable equilibrium state for the system (16).

As shown in section, when the condition (15) is satisfied, the reaction rate for the reduced system (5) is *c* = *λ*_*n*_. But how do we calculate *λ*_*n*_? Let *β* denote the solution for (29) and *γ* = −*f*_*n*_(1−**1** ^*T*^ *β*). When condition (23) is satisfied, *λ*_*n*_ can be well approximated by *γ* in terms of Bauer and Fike theorem [17] and Corollary 2.2 in Eisenstat and Ipsen [18] (see S1 Appendix) with a relative error

$$\frac{|\lambda_{n}-\gamma |}{|\lambda_{n}|}=O\left(f_{n}T_{relax}\right).$$

### General Case

Now we can loosen the condition that there is only one particle in the system (4). Suppose there are totally *m* particles. Then each particle has its own exit time *T*_*j*_, *j* = 1, …, *m*. The earliest exit time *T*_0_ is the minimum of all exit times. *T*_0_ is of concern because it represent the first time one of the particles will exit from the system and may lead to significant event that is not included in the system. To formulate it, we have

$$\begin{array}{c} T_{0}=\min_{1\leq j\leq m}T_{j}. \end{array}$$ (30)

We have

$$\begin{array}{c} \text{Prob}\left(T_{0}\geq t\right)=\text{Prob}(T_{j}\geq t,\;\text{for}\;\text{all}\;1\leq j\leq m)=\prod_{j=1}^{m}\text{Prob}\left(T_{j}\geq t\right). \end{array}$$ (31)

When condition (23) is satisfied, as shown in previous section, for each *j*, the corresponding exit time *T*_*j*_ well approximates an exponential distribution with the same rate *λ*_*n*_. Thus we have

$$\begin{array}{c} \text{Prob}\left(T_{j}\geq t\right)\approx e^{\lambda_{n}t}, \end{array}$$ (32)

and

$$\begin{array}{c} \text{Prob}\left(T_{0}\geq t\right)=\prod_{j=1}^{m}\text{Prob}\left(T_{j}\geq t\right)\approx e^{m\lambda_{n}t}, \end{array}$$ (33)

Thus for system (4) with *m* particles, when condition (23) is satisfied, it can be reduced to system (5) with reaction rate *c* = *m*∣*λ*_*n*_∣.

#### Remark

- *A* does not have to be a constant matrix. The above analysis is based on a linear chain reaction system of fast variables but not necessary of slow variables. Between slow reaction firings, slow variables do not change. The reaction rates may be nonlinear functions of slow variables. In that case, the eigenvalues of the matrix *A* will change with slow reaction firings. This will be the case in our numerical experiments.
- The analysis can be easily extended to a system that is slightly different from (4). For example, one can change the system to
  $$\begin{array}{c} S_{1}\overset{f_{1}}{\underset{b_{1}}{⇌}}S_{2}\overset{f_{2}}{\underset{b_{2}}{⇌}}\cdots \overset{f_{n-1}}{\underset{b_{n-1}}{⇌}}S_{n}, \end{array}$$
  and
  $$\begin{array}{c} S_{i}\overset{f_{n}}{\to }S_{n+1}, \end{array}$$
  where *i* could be any integer between 1 and *n*. It is easy to verify that our analysis is still valid. The only difference is where the extra diagonal term *f*_*n*_ is located. Furthermore, we will show in S2 Appendix that our analysis can also be extended to systems like
  $$\begin{array}{c} \begin{array}{c} S_{1}\overset{E}{\underset{k_{0}}{⇌}}S_{2}\overset{E}{\underset{k_{0}}{⇌}}\cdots \overset{E}{\underset{k_{0}}{⇌}}S_{n},\\ \emptyset ⟶E\overset{k_{u}S_{u}}{\to }\emptyset , \end{array} \end{array}$$ (34)
  where the chain reactions are fast and the degradation reaction for *E* is slow. Here *E*, as an enzyme, does not change its value in the chain reaction, and each individual forward reaction has the form
  $$\begin{array}{c} S_{i}+E⟶S_{i+1}+E,\;\text{for}\;i=1,\cdots ,n-1, \end{array}$$
  with elementary reaction rate laws, while *S*_*i*_’s do not change values in the enzymic reactions
  $$\begin{array}{c} E+S_{i}⟶S_{i}, \end{array}$$
  either.

## Results

In this section we test the accuracy of abridgment with a bistable switch model consisting of a chain reaction system. Numerical experiments on two oscillation models based on this bistable switch model are also presented. These simulations were performed on a 3.0GHz Intel Core Linux workstation.

### Bistable Switch Model

This is the bistable switch model in Fig 2. We rewrite it as

$$\begin{array}{c} \begin{array}{c} Cdh1P_{0}\overset{k*Clb2}{\underset{k*k_{p}}{⇌}}Cdh1P_{1}\overset{k*Clb2}{\underset{k*k_{p}}{⇌}}\cdots \overset{k*Clb2}{\underset{k*k_{p}}{⇌}}Cdh1P_{9},\\ \emptyset \overset{k_{s}}{\to }Clb2,\\ Clb2\overset{k_{a}\sum_{i=0}^{9}\left(10-i\right)Cdh1P_{i}}{\to }\emptyset . \end{array} \end{array}$$ (35)

where *k*_*s*_ and *k*_*a*_ are constants corresponding to Clb2’s synthesis and degradation. *k* is a scalar that controls the reaction scale of the chain system. These enzymic reactions all follow elementary reaction rate laws. Note that our analysis can be applied to this system (see S2 Appendix) although the format appears different than (4). We will test the accuracy when this system is reduced to

$$\begin{array}{c} \begin{array}{c} \emptyset \overset{k_{s}}{\to }Clb2,\\ Clb2\overset{Cdh1*\lambda }{\to }\emptyset , \end{array} \end{array}$$ (36)

where Cdh1 is the total population of Cdh1P_i_ and *λ* is calculated accordingly. By intuition, when *k* is large, all reactions in the linear chain system are fast, the quasi steady state assumption is applicable and as a result the reduction is valid. By contrast, if *k* is so small that propensities of chain reactions are comparable to the two slow reactions, great errors will be introduced.

**Fig 3:**
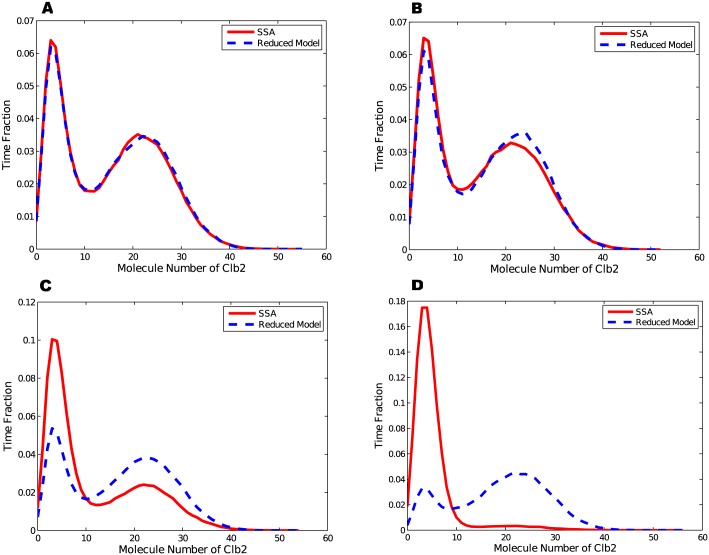
Distributions of Clb2 time fraction from original model and reduced model. (A)*k* = 5 and the distribution density error is 4.53%. (B)*k* = 0.5 and the distribution density error is 7.81%. (C)*k* = 0.05 and the distribution density error is 51.7%. (D)*k* = 0.01 and the distribution density error is 147%.

Accuracy of the reduction is studied with four groups of numerical experiments. Let *k*_*p*_ = 8.0, *k*_*a*_ = 5.5 × 10^−4^, *k*_*s*_ = 350*k*_*a*_, and the total population of Cdh1 and the initial value of Clb2 be 10. *k* is chosen to be 5, 0.5, 0.05 and 0.01 respectively. In each experiment the system is simulated till 10^6^ time units. Distributions of Clb2 are shown in Fig 3.

**Fig 4:**
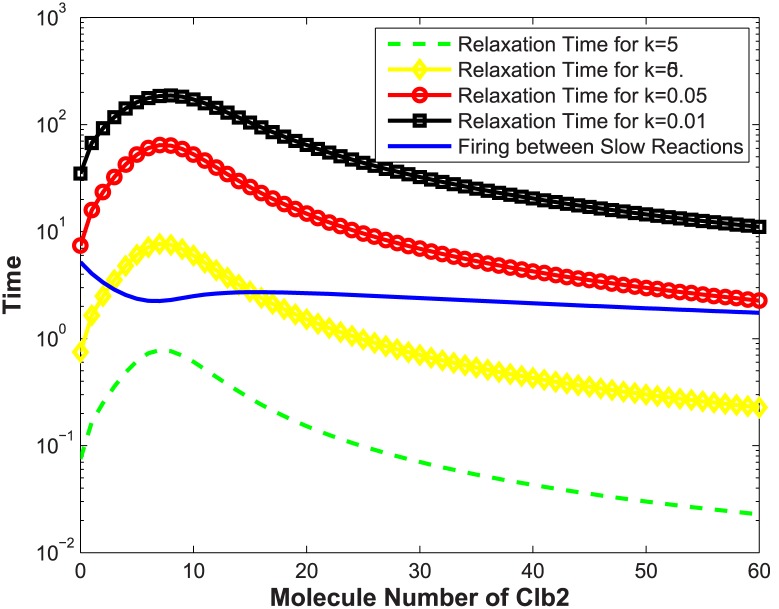
Relaxation time of the chain reaction system. Relaxation time and time interval between two slow reaction events are computed with different parameters of *k* and molecule numbers of Clb2.

Relaxation time of the chain reaction in this model can be calculated as a function of the parameter *k* and the population of Clb2. Theoretical results with respect to *k* along with different Clb2 are given in Fig 4. The curve describing time intervals between two slow reaction firings is also plotted.

When *k* = 5, the dashed green curve (for *k* = 5) in Fig 4 is below the solid curve. Thus the chain reaction system reaches steady states quickly before Clb2 gets updated. The condition (23) holds and the reduction is valid (see Fig 3(A)); When *k* = 0.5, the diamond yellow curve (for *k* = 0.5) has two intersections with the solid curve at about Clb2 = 5 and Clb2 = 15. When Clb2 stays within this range, population of Clb2 changes slightly faster than the chain reaction system. In the bistable switch model, Clb2 changes between two steady states and has to cross this region ([5, 15]), within which the condition (23) does not hold. The time distribution in Fig 3(B) shows mismatch to some extent; When *k* = 0.05, in Fig 4, the dotted red curve (for *k* = 0.05) is always above the solid curve. The condition (23) is not satisfied. In Fig 3(C), the time distribution given by the reduced model shows much greater errors, although bistable states are still observable; When *k* reduces to 0.01, the squared black curve (for *k* = 0.01) shows much larger relaxation times for the chain reaction system, well above the solid blue curve. The condition (23) is broken, and errors from the reduction result in difference in system characteristics (Fig 3(D)). The original model (solid red curve) has only one stable state, but the reduced model shows two stable states.

Meanwhile, CPU times for the original model and for the reduced model are shown in Table 1. We can see that the CPU times of the original model are proportional to the value of *k*, while the CPU times of the reduced model are approximately the same. When *k* = 5, the simulation on the reduced model is at least 1,000 times faster than on the original model.

**Table 1:** CPU time comparison on the bistable switch model.

k	Original model	Reduced Model
5	157.98s	0.0441s
0.5	15.62s	0.0456s
0.05	1.46s	0.0442s
0.01	0.48s	0.0454s

To sum up, the abridgment is valid for this model when the condition (23) is satisfied. In this case the abridgment shows much better efficiency and reasonable accuracy compared with the original model. However, if (23) is not satisfied, abridgment may cause large errors with little efficiency gain. Particularly if the relaxation time of the chain reaction system is much larger than the mean time for slow reactions, the error could be so large that important system characteristics may get changed.

### Oscillation Models

Biochemical oscillations occur in many biological systems. The above bistable switch is an important module in biochemical oscillations. To test the abridgment on oscillation models, we construct two oscillation models based on the Clb2 bistable switch model. We introduce a third reactant *z* and design negative feedback loops involving two or three of the species. Numerical experiments for abridgment are analyzed as follows.

#### Oscillation Model One

**Fig 5:**
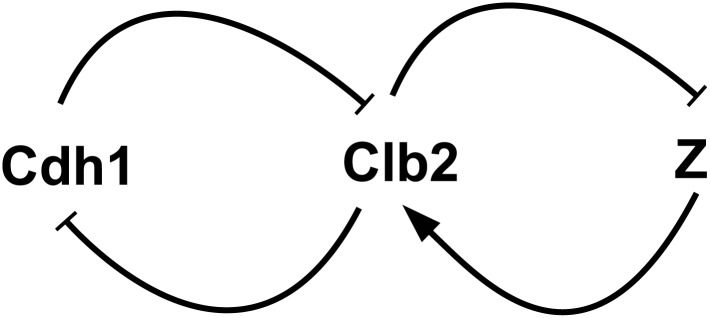
Motif of oscillation model one. In this motif, Clb2 and Cdh1 form a positive feedback loop and Clb2 and Z construct a negative feedback loop.

**Fig 6:**
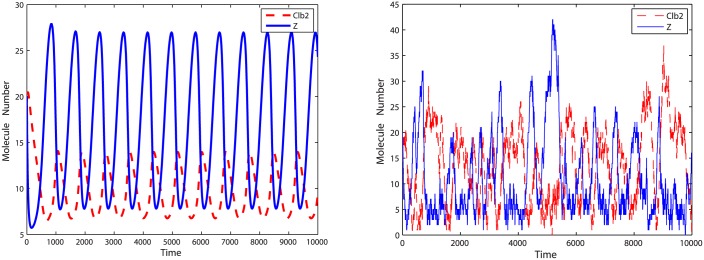
Deterministic and stochastic simulation results of oscillation model one. Phase property of oscillations in stochastic simulation consists of what shows in deterministic simulation. Amplitude of Clb2 is enlarged due to stochastic noise compared with amplitude in deterministic simulation. **Left**: Deterministic simulation results. **Right**: Stochastic simulation results.

The first oscillation model consists a negative feedback loop in terms of Clb2 and Z. The interaction motif is given in Fig 5, where Clb2 ⊣ Z means “Clb2 inhibits Z” and Z → Clb2 means “Z actives Clb2”. Detailed reactions are given in S3 Appendix, and the oscillations of Clb2 and Z are plotted in Fig 6.

**Fig 7:**
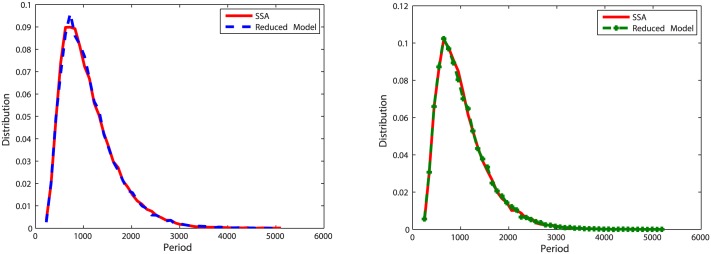
Distributions of oscillation periods of Clb2 and Z in oscillation model one with *k* = 5. **Left**: Plots for Clb2. The distribution density error is 4%. **Right**: Plots for Z. The distribution density error is 3.3%.

**Fig 8:**
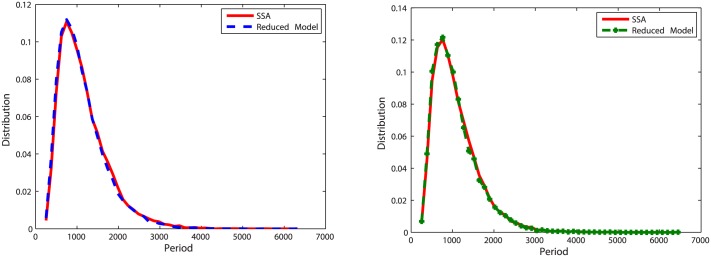
Distribution of oscillation periods of Clb2 and Z in oscillation model one with *k* = 0.5. **Left**: Plots for Clb2. The distribution density error is 4.4%. **Right**: Plots for Z. The distribution density error is 3.9%.

**Fig 9:**
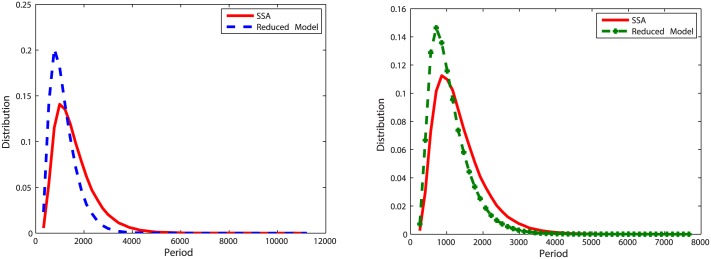
Distribution of oscillation periods of Clb2 and Z in oscillation model one with *k* = 0.05. **Left**: Plots for Clb2. The distribution density error is 47.57%. **Right**: Plots for Z. The distribution density error is 33.27%.

With initial values Clb2 = 20, Cdh1 = 10 and Z = 20, we run simulations for *k* = 5, *k* = 0.5 and *k* = 0.05 (when *k* = 0.01, there is no oscillation in the deterministic model (Figure A in S3 Appendix), thus we did not simulate this case in the oscillation model). To measure the accuracy of the reduced model, we recorded 20,000 oscillation periods of Clb2 and Z and compared the distribution of oscillation periods with results simulated with the original model. Corresponding results are plotted in Figs 7, 8 and 9.

When *k* = 5, the reduced model almost has the same behavior as the original model. When *k* decreases to 0.5, we can see in Fig 8, the distribution density errors for Clb2 and Z are close to the results of the case *k* = 5. When *k* = 0.05, the difference is obvious.

#### Oscillation Model Two

**Fig 10:**
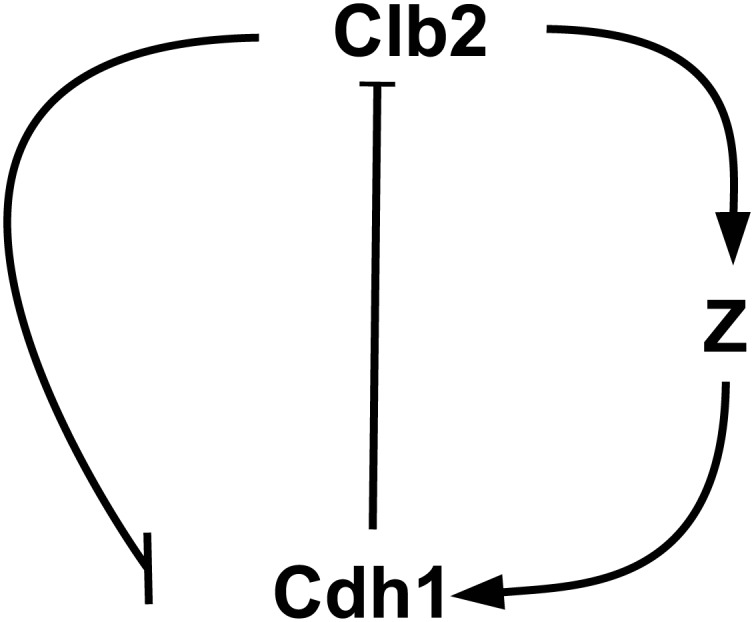
Motif of oscillation model two. In this motif, Clb2 and Cdh1 form a positive feedback loop. Cdh1, Clb2 and Z construct a negative feedback loop.

**Fig 11:**
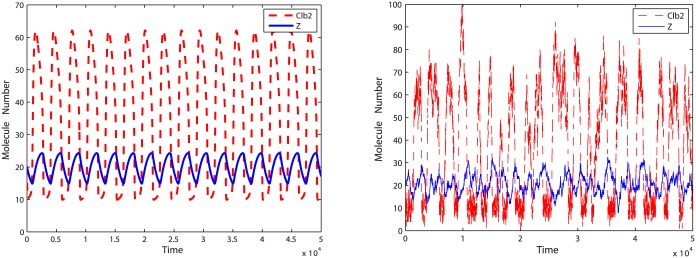
Simulation results of oscillation model two. **Left**: Deterministic simulation results. **Right**: Stochastic simulation results.

For the second oscillation model, the motif is given in Fig 10. The simulation results of Clb2 and Z oscillation are plotted in Fig 11. Corresponding reactions are described in S4 Appendix. Different from previous models, in this chain reaction system, the dephosphorylation reaction rate is not a constant. Z here acts as an enzyme activating the phosphorylation of Cdh1.

**Fig 12:**
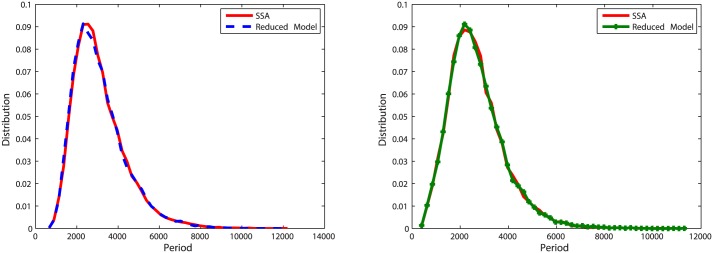
Distribution of oscillation periods of Clb2 and Z in oscillation model two with *k* = 5. **Left**: Plots for Clb2. The distribution density error is 3.73%. **Right**: Plots for Z. The distribution density error is 3.66%.

**Fig 13:**
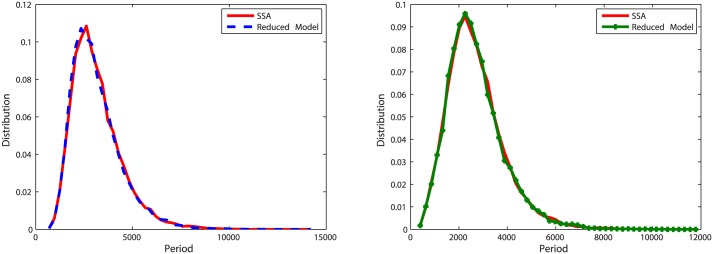
Distribution of oscillation periods of Clb2 and Z in oscillation model two with *k* = 0.5. **Left**: Plots for Clb2. The distribution density error is 6.06%. **Right**: Plots for Z. The distribution density error is 4.33%.

**Fig 14:**
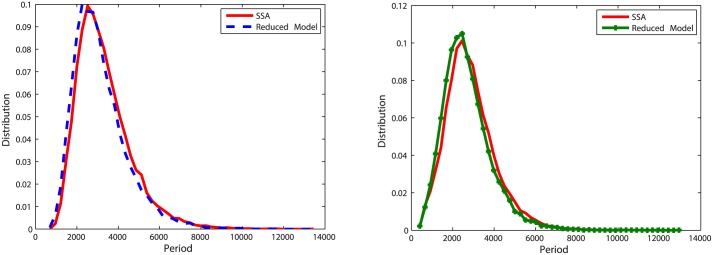
Distribution of oscillation periods of Clb2 and Z in oscillation model two with *k* = 0.05. **Left**: Plots for Clb2. The distribution density error is 13.2%. **Right**: Plots for Z. The distribution density error is 13.8%.

With initial values of Clb2 = 20, Cdh1 = 10 and Z = 20, we run simulations for *k* = 5, *k* = 0.5 and *k* = 0.05. To measure the accuracy of the reduced model, 20,000 oscillation periods of Clb2 and Z are recorded. The distribution of oscillation periods for the reduced model is compared with that for the original model in Figs 12, 13 and 14. Similar with the pattern shown in oscillation model one, when *k* = 5 and *k* = 0.5 the abridgment gives great approximation on the oscillation model. Larger error comes from *k* = 0.05, where the distribution density errors are about 10%.

To check the efficiency of abridgment of these two oscillation model, CPU times are given in Table 2. The CPU time for the original model is approximately proportional to the value of parameter *k* for both models. But the CPU times for the reduced model are close. For the first oscillation model, abridgment shows advantage for all three cases. However, for the second model, when *k* = 0.05, the CPU time for the reduced model is almost the same as that for the original time.

**Table 2:** Algorithm execution time comparison on oscillation models.

k	Oscillation Model One		Oscillation Model Two	
	Original Model	Reduced Model	Original Model	Reduced Model
5	87946s	67.60s	41766s	318.47s
0.5	8738s	67.19s	3821s	369.93s
0.05	1274s	65.07s	421s	364.95s

## Conclusion and Discussion

In this paper we present a theoretical analysis for the abridgment from a chain reaction system to a simple reaction. Starting from the CME of a simple particle, we demonstrate that the condition for the abridgment is related to the scale difference between the relaxation time and the mean reaction time for slow reactions. Our analysis shows that if the relaxation time of the fast subsystem is much smaller than the mean firing time of the slow reaction, the abridgment can be applied with little errors. Numerical experiments on a bistable switch demonstrate that when this condition is broken, great errors may appear. On the other hand, we test the abridgment on two biochemical oscillation models based on the same bistable switch module. We can see that even though there could be greater errors on the bistable switch module resulted from abridgment, the overall results for these two oscillation models could still be close. There might be other system properties that help to reduce the errors caused by the abridgment. Further study is still needed to study what these system properties are.

## Supporting Information

S1 Appendix Reaction rate for the reduced system. (PDF)

S2 Appendix Application of the abridgment to bistable switch model. (PDF)

S3 Appendix Details for model one. (PDF)

S4 Appendix Details for model two. (PDF)

## References

[1] Gillespie D. A general method for numerically simulating the stochastic time evolution of coupled chemical reactions. J Comput Phys. 1976;22:403–434. 10.1016/0021-9991(76)90041-3

[2] Gillespie D. Exact stochastic simulation of coupled chemical reactions. J Phys Chem. 1977;81:2340–2361. 10.1021/j100540a008

[3] Gillespie DT, Cao Y, Sanft KR, and Petzold LR. The subtle business of model reduction for stochastic chemical kinetics. J Chem Phys. 2009;130:064103 10.1063/1.3072704 19222263 PMC2675560

[4] Kar S, Baumann W, Paul M, Tyson J. Exploring the role of noise in the eukaryotic cell cycle. PNAS. 2009;106:6471–6476. 10.1073/pnas.0810034106 19246388 PMC2672517

[5] Qu Z, Weiss J, MacLellan W. Regulation of the mammalian cell cycle: a model of the G1-to-S transition. Am J Physiol Cell Physiol. 2003;284:C349C364 10.1152/ajpcell.00066.2002 12388094

[6] Kapuy O, Barik D, Sananes M, Tyson J, Novak B. Bistability by multiple phosphorylation of regulatory protein. Prog biophys mol biol. 2005;. 10.1016/j.pbiomolbio.2009.06.004 PMC2784190 19523976

[7] Barik D, Baumann WT, Paul MR, Novak B, Tyson JJ. A model of yeast cell-cycle regulation based on multisite phosphorylation. MOLECULAR SYSTEMS BIOLOGY. 2010 8;6 10.1038/msb.2010.55 20739927 PMC2947364

[8] Di Talia S, Skotheim JM, Bean JM, Siggia ED, Cross FR. The effects of molecular noise and size control on variability in the budding yeast cell cycle. NATURE. 2007 8 23;448(7156):947–U12. 10.1038/nature06072 17713537

[9] Rao C, Arkin A. Stochastic chemical kinetics and the quasi steady-state assumption. J Chem Phys. 2003;118:4999–5010. 10.1063/1.1545446

[10] Cao Y, Gillespie D, Petzold L. The slow-scale stochastic simulation algorithm. J Chem Phys. 2005;122:014116 10.1063/1.1824902 15638651

[11] Cao Y, Gillespie D, Petzold L. Multi-scale stochastic simulation algorithm with stochastic partial equilibrium assumption for chemically reacting systems. J Comput Phys. 2005;206:395–411. 10.1016/j.jcp.2004.12.014

[12] Thomas P, Straube AV, Grima R. Communication: Limitations of the stochastic quasi-steady-state approximation in open biochemical reaction networks. J Chem Phys. 2011;135 10.1063/1.3661156 22088045

[13] Thomas P, Straube AV, Grima R. Communication: Limitations of the stochastic quasi-steady-state approximation in open biochemical reaction networks. The Journal of Chemical Physics. 2011;135(18):–. Available from: http://scitation.aip.org/content/aip/journal/jcp/135/18/10.1063/1.3661156 10.1063/1.3661156 22088045

[14] Ledermann W, Reuter GEH. Spectral Theory for the Differential Equations of Simple Birth and Death Processes. Philosophical Transactions of the Royal Society of London Series A, Mathematical and Physical Sciences. 1954;246:321–369. 10.1098/rsta.1954.0001

[15] Kijima M. Markov Processes for Stochastic Modeling. Chapman and Hall/CRC; 1997.

[16] Wilkinson JH. The Algebraic Eigenvalue Problem. Oxford University Press; 1965.

[17] Bauer FL, Fike CT. Norms and exclusion theorems. Numer Math. 1960;2:137–141. 10.1007/BF01386217

[18] Eisenstat SC, Ipsen ICF. Three absolute perturbation bounds for matrix eigenvalues imply relative bounds. SIAM Journal on Matrix Analysis and Applications. 1998;20:149–158. 10.1137/S0895479897323282

