# Supplementary material for: The Abridgment and Relaxation Time for a Linear Multi-Scale Model Based on Multiple Site Phosphorylation
Source: PLoS One. 2015 Aug 11;10(8):e0133295. doi: 10.1371/journal.pone.0133295 (PMC4532472; doi:10.1371/journal.pone.0133295)
Supplement: S1 Appendix — (PDF) [file pone.0133295.s001.pdf]

S1 Appendix. Reaction rate for the reduced system.

Let

$$L = \begin{bmatrix} I & 0 \\ -\mathbf{1}^T & 1 \end{bmatrix}, \tag{1}$$

where I is the identity matrix of dimension $n-1$. Then with a similarity transformation by L we have

$$L^{-1}AL = \begin{bmatrix} \tilde{B} & y \\ f_n\mathbf{1}^T & -f_n \end{bmatrix}. \tag{2}$$

Let β denote the solution for equation (29). We construct a nonsingular matrix

$$M = \begin{bmatrix} I & \beta \\ 0 & 1 \end{bmatrix}. \tag{3}$$

Continue the similarity transformation by M, we have

$$\mathbb{A} = M^{-1}L^{-1}ALM = \begin{bmatrix} \tilde{B} - f_n\beta\mathbf{1}^T & f_n(1-\mathbf{1}^T\beta)\beta \\ f_n\mathbf{1}^T & -f_n(1-\mathbf{1}^T\beta) \end{bmatrix}. \tag{4}$$

When $f_n \ll 1/T_{relax}$, f_n is small. Let $\gamma = -f_n(1-\mathbf{1}^T\beta)$ and

$$\bar{A} = \begin{bmatrix} \tilde{B} - f_n\beta\mathbf{1}^T & 0 \\ f_n\mathbf{1}^T & \gamma \end{bmatrix}. \tag{5}$$

Then the eigenvalues of A can be approximated by corresponding eigenvalues of $\bar{A}$. Note that γ is an eigenvalue of $\bar{A}$ and $|\gamma| < f_n$, according to (23) $|\lambda_n| < f_n \ll \min_{1\le i\le n-1} |\hat{\lambda}_i|$. Thus γ approximates λ_n.

We denote the perturbation matrix as

$$E = \mathbb{A} - \bar{A} = \begin{bmatrix} 0 & -\gamma\beta \\ 0 & 0 \end{bmatrix}. \tag{6}$$

From the Bauer and Fike theorem [14], we have that

$$|\lambda_n - \gamma| \leq \|E\| \leq |\gamma|. \tag{7}$$

This estimation allows a large relative error. To accurately estimate the relative error, we note that $\mathbb{A}$ is similar to A, which is diagonalizable. Then according to Corollary 2.2 in Eisenstat and Ipsen [15], we have

$$\frac{|\lambda_n - \gamma|}{|\lambda_n|} \leq \|\mathbb{A}^{-1}E\| = \left\| \begin{bmatrix} 0 & -\gamma\tilde{B}^{-1}\beta \\ 0 & f_n\mathbf{1}^T\tilde{B}^{-1}\beta \end{bmatrix} \right\| = O\left(\frac{f_n}{\min_{1\leq i\leq n-1}|\hat{\lambda}_i|}\right) = O(f_nT_{relax}). \tag{8}$$

Thus when condition (23) is satisfied, λ_n can be well approximated by γ.
