# Supplementary material for: The Abridgment and Relaxation Time for a Linear Multi-Scale Model Based on Multiple Site Phosphorylation
Source: PLoS One. 2015 Aug 11;10(8):e0133295. doi: 10.1371/journal.pone.0133295 (PMC4532472; doi:10.1371/journal.pone.0133295)
Supplement: S2 Appendix — (PDF) [file pone.0133295.s002.pdf]

S2 Appendix. Application of the abridgment to bistable switch model.

In the Methods section, we mainly analyzed the linear chain system (4). The technique can also be applied to a general case shown as

$$\begin{aligned} &S_1 \xrightleftharpoons[b_1]{f_1} S_2 \xrightleftharpoons[b_2]{f_2} \cdots \xrightleftharpoons[b_{n-1}]{f_{n-1}} S_n, \\ &S_u \xrightarrow{f_n} S_{n+1}, \qquad \text{with } u \in \{1, 2, \ldots, n\}. \end{aligned} \tag{1}$$

The matrix A defined in (14) under such condition is a little different. We introduce a n-by-n matrix E_u. E_u has all the elements equal to 0, expect $E_{uu} = -f_n$. Therefore, matrix A is written as

$$A = B + E_u,$$

where B is the matrix defined in (19) for the fast subsystem. We can obtain the same conclusion for the linear chain system with different u.

In the Results section, we present a bistable switch model as a system (35). In this section, we will show that this system is a combination of multiple linear chain systems as (1). In system (35), we assume proteins $Cdh1P_i$, $i = 0, \ldots, 9$ form a fast linear chain subsystem. At the same time, $Cdh1P_i$ plays a role as an enzyme activating the $Clb2$ degradation. For each $Cdh1P_u$, model (35) can be represented as

$$\begin{aligned} &Cdh1P_0 \xrightleftharpoons[k * k_p]{k * Clb2} Cdh1P_1 \xrightleftharpoons[k * k_p]{k * Clb2} \ldots \xrightleftharpoons[k * k_p]{k * Clb2} Cdh1P_9, \\ &\emptyset \xrightarrow{k_s} Clb2, \\ &Clb2 \xrightarrow{k_a(10-i)Cdh1P_u} \emptyset. \end{aligned} \tag{2}$$

Note that synthesis and degradation of $Clb2$ are considered as slow reactions. Between successive slow reactions, population of $Clb2$ doesn't change. By ignoring the population change of $Clb2$, for each $Cdh1P_u$ we can see that the system has the same

pattern as (34).

$$S_1 \underset{k_0}{\overset{\text{E}}{\rightleftharpoons}} S_2 \underset{k_0}{\overset{\text{E}}{\rightleftharpoons}} \ldots \underset{k_0}{\overset{\text{E}}{\rightleftharpoons}} S_n,$$

$$\emptyset \longrightarrow E \xrightarrow{k_u S_u} \emptyset,$$

We would like to show that our analysis can be applied to this type of system, and (34) can be simply reduced to

$$E \xrightarrow{\lambda_u S_{\text{total}}} \emptyset, \tag{3}$$

where S_{total} represents the total population of S. Note the difference between (34) and (4) is that in (34), the total population of S does not change in the slow reaction, while in (4), every time the slow reaction fires, one particle will leave the fast subsystem and becomes S_{n+1}. However, the analysis still can be applied. We just need to define proper state variables and present the proper CMEs. Assume there is one particle in S and one particle in E at the beginning, we define

$$p_i(t) = \text{Prob}(S_i(t) = 1, E(t) = 1), \quad \text{for} \quad i = 1, \ldots, n, \tag{4}$$

and $p_{n+1}(t) = \text{Prob}(E(t) = 0)$ and let $\mathbb{P}(t) = (p_1(t), \ldots, p_n(t))^T$. Note that in this case, we still have

$$\sum_{i=1}^{n+1} p_i(t) = 1. \tag{5}$$

All the derivation in our analysis follows. Then for each u, we have that the first exit time for the slow reaction follows an exponential distribution with rate $\lambda_u S_{total}$, if we consider the combination of them, we conclude that the whole system can be reduced to

$$E \xrightarrow{\lambda S_{\text{total}}} \emptyset, \tag{6}$$

where $\lambda = \sum_{i=1}^{n} \lambda_u$, and that is the reduced model (36).
