# Supplementary material for: The Abridgment and Relaxation Time for a Linear Multi-Scale Model Based on Multiple Site Phosphorylation
Source: PLoS One. 2015 Aug 11;10(8):e0133295. doi: 10.1371/journal.pone.0133295 (PMC4532472; doi:10.1371/journal.pone.0133295)
Supplement: S3 Appendix — (PDF) [file pone.0133295.s003.pdf]

S3 Appendix. Details for model one.

$$
\begin{aligned}
&Cdh1P_0 \underset{k*k_p}{\overset{k*Clb2}{\rightleftharpoons}} Cdh1P_1 \underset{k*k_p}{\overset{k*Clb2}{\rightleftharpoons}} \dots \underset{k*k_p}{\overset{k*Clb2}{\rightleftharpoons}} Cdh1P_9,\\
&\emptyset \xrightarrow{k_{xs}} Clb2,\\
&Clb2 \xrightarrow{k_{xd}} \emptyset,\\
&\emptyset \xrightarrow{k_{zs}} Z,\\
&Z \xrightarrow{k_{zd}} \emptyset,
\end{aligned}
\tag{1}
$$

where the synthesis and degradation rates of Clb2 and Z are

$$
\begin{aligned}
&k_{xs} = k_a * (k_{xs1} + k_{xs2} * (0.1 + 0.9\tfrac{Z^m}{13^m + Z^m})),\\
&k_{xd} = k_a * (\textstyle\sum_{i=0}^{9}(10-i)Cdh1P_i),\\
&k_{zs} = k_c * k_b,\\
&k_{zd} = k_c * (0.1 + 0.9\tfrac{Clb2^n}{12^n + Clb2^n}).
\end{aligned}
$$

The values of corresponding parameters are given in Table A.

Parameter	Value	Parameter	Value
k_p	8.0	k_b	5.0
k_a	5.5e-4	k_c	0.03
k_{xs1}	222	n	5
k_{xs2}	278	m	3

Table A. Parameters of chemical reactions in oscillation model one.

If the chain reactions are fast reactions, with their relaxation time smaller than the

mean firing time of other four reactions, this oscillation model is reduced into

$$\begin{aligned}
\emptyset &\xrightarrow{k_{xs}} Clb2, \\
Clb2 &\xrightarrow{Cdh1*\lambda} \emptyset, \\
\emptyset &\xrightarrow{k_{zs}} Z, \\
Z &\xrightarrow{k_{zd}} \emptyset,
\end{aligned} \tag{2}$$

where $Cdh1 = \sum_{i=0}^{9} Cdh1P_i$ and λ is the absolute value of the largest eigenvalue of corresponding matrix derived from the chain reaction system.

Deterministic simulation results are shown in Fig. A. Clb2 oscillation of reduced model matches well with that of the complete model when k is large $(= 5)$. Simulation results show obvious divergence when k reduces to 0.01. There is no oscillation in the complete model while oscillation in the reduced model remains.

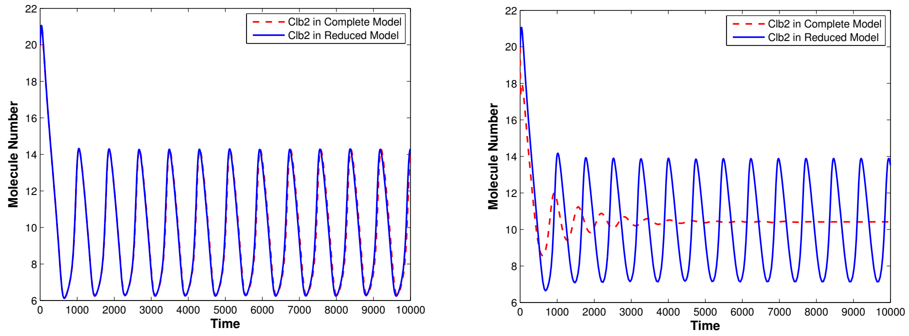

Fig A. Oscillations of Clb2 in model one and the corresponding reduced model. Two models show the same behavior under the case of $k = 5$. When k reduces to 0.01, the reduced model still has a stable oscillation of Clb2. However, oscillation of Clb2 in the complete model vanishes. **Left**: Plots for Clb2 in model one and reduced model with respect to $k = 5$. **Right**: Plots for Clb2 in model one and reduced model with respect to $k = 0.01$.
