# Supplementary material for: The Abridgment and Relaxation Time for a Linear Multi-Scale Model Based on Multiple Site Phosphorylation
Source: PLoS One. 2015 Aug 11;10(8):e0133295. doi: 10.1371/journal.pone.0133295 (PMC4532472; doi:10.1371/journal.pone.0133295)
Supplement: S4 Appendix — (PDF) [file pone.0133295.s004.pdf]

S4 Appendix. Details for model two.

$$
\begin{aligned}
Cdh1P_0 \underset{k*Z}{\overset{k*Clb2}{\rightleftharpoons}} Cdh1P_1 \underset{k*Z}{\overset{k*Clb2}{\rightleftharpoons}} \ldots \underset{k*Z}{\overset{k*Clb2}{\rightleftharpoons}} Cdh1P_9,\\
\emptyset \xrightarrow{k_{xs}} Clb2,\\
Clb2 \xrightarrow{k_{xd}} \emptyset,\\
\emptyset \xrightarrow{k_{zs}} Z,\\
Z \xrightarrow{k_{zd}} \emptyset,
\end{aligned}
\tag{1}
$$

where the synthesis and degradation rates of Clb2 and Z are

$$
\begin{aligned}
k_{xs} &= k_a * kxr,\\
k_{xd} &= k_a * (\textstyle\sum_{i=0}^{9}(10-i)Cdh1P_i),\\
k_{zs} &= k_c * (10 + 20\frac{Clb2^n}{30^n + Clb2^n}),\\
k_{zd} &= k_c * k_b.
\end{aligned}
$$

And corresponding parameters are shown in Table A. The reduced model based on the

Parameter	Value	Parameter	Value
k_a	5.5e-4	kxr	910
k_b	1	n	3
k_c	0.001		

Table A. Parameters of chemical reactions in oscillation model two.

fast reaction assumption has the same format as model in S3 Appendix, but with different reaction rate constants.

Deterministic simulation results are plot in Fig. A. Oscillation in Model two exists with a wider range of k. The reduced model converges to steady state when $k = 1.5e-3$ while the complete model still shows oscillations.

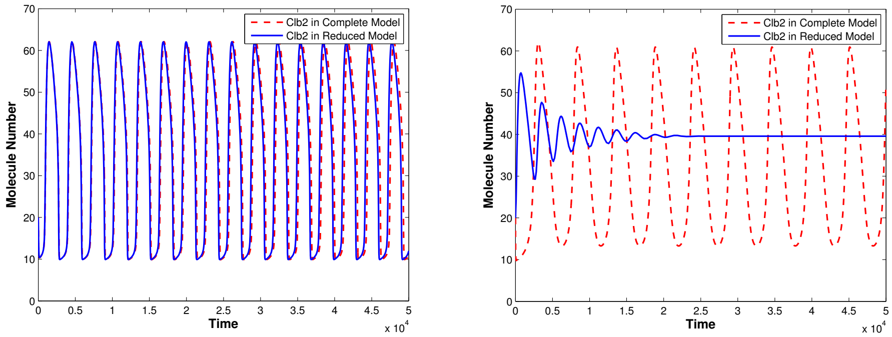

Fig A. Oscillations of Clb2 in model two and corresponding reduced model. Mismatch of Clb2 oscillations becomes clear as k decreases from 0.5 to $1.5e-3$. Reduced model loses its oscillation property at $k = 1.5e-3$, while the complete model also shows Clb2 oscillation although with a larger period compared to period with respect to $k = 0.5$. **Left**: Plots for Clb2 in model two and reduced model with respect to $k = 0.5$. **Right**: Plots for Clb2 in model two and reduced model with respect to $k = 1.5e-3$.
